# Maternal Serum Homocysteine as a Severity-Linked Biomarker Across the Spectrum of Hypertensive Disorders of Pregnancy: A Prospective Observational Study

**DOI:** 10.7759/cureus.109335

**Published:** 2026-05-21

**Authors:** Mouli Nandi, Rana Mondal

**Affiliations:** 1 Department of Obstetrics and Gynaecology, Luton and Dunstable University Hospital, Luton, GBR

**Keywords:** gestational hypertension, homocysteine, postpartum eclampsia, severe hypertension, severe preeclampsia

## Abstract

Background: Hypertensive disorders of pregnancy (HDP) remain a leading cause of maternal and perinatal morbidity worldwide. Endothelial dysfunction is central to the pathogenesis of preeclampsia, and serum homocysteine, a pro-oxidant amino acid, has been implicated in vascular injury and placental insufficiency.

Methods: This prospective observational study included 200 pregnant women beyond 20 weeks of gestation, stratified into four equal groups: normotensive controls, non-severe preeclampsia, severe preeclampsia, and eclampsia (n = 50 each). Serum homocysteine levels were measured at admission prior to therapeutic intervention. Clinical and demographic variables were recorded. Continuous variables were analysed using one-way ANOVA and categorical variables using the χ² test, with statistical significance set at p < 0.05.

Results: Maternal serum homocysteine levels demonstrated a significant and progressive increase with disease severity, rising from 12.04 ± 2.34 µmol/L in normotensive women to 26.74 ± 6.58 µmol/L in eclampsia (p = 0.0251). Primigravidity was more common in severe disease groups (p = 0.0375), and maternal body mass index increased with severity (p = 0.0461). Caesarean section rates were significantly higher in severe preeclampsia (70%) and eclampsia (80%) compared with controls (p < 0.001). Blood pressure normalised across all groups at 12 weeks postpartum.

Conclusions: Maternal serum homocysteine is strongly associated with both the presence and severity of HDP. Its progressive elevation across the disease spectrum highlights its potential role as a clinically useful biomarker for early risk stratification and management. Integration of homocysteine assessment into routine obstetric care may enhance the prediction of severe disease and improve maternal outcomes.

## Introduction

Hypertensive disorders of pregnancy are common and clinically consequential, affecting approximately 5-10% of pregnancies worldwide [1]. International classification and management guidance is provided by bodies such as the American College of Obstetricians and Gynecologists (ACOG) [2] and the International Society for the Study of Hypertension in Pregnancy (ISSHP) [3,4]. While diagnostic criteria have evolved (notably, proteinuria is not mandatory in all modern definitions), the shared clinical trajectory is progressive maternal vascular dysregulation with potential for rapid decompensation into severe features and eclampsia [5].

The endothelial dysfunction hypothesis provides a coherent mechanistic framework for the maternal syndrome. In updated placental stress models (e.g., Staff [6]), syncytiotrophoblast stress and shedding are central features across early- and late-onset phenotypes. In the influential two-stage model articulated by Roberts et al., a poorly perfused or stressed placenta (Stage 1) drives the maternal clinical syndrome (Stage 2) through circulating mediators that induce systemic endothelial dysfunction and inflammation [7]. Subsequent refinements from Redman et al. and others emphasise that multiple "placental causes" and maternal constitutional susceptibility can converge on the same final common pathway - maternal vascular injury with capillary leak, vasoconstriction, and prothrombotic change [8].

Evidence connecting homocysteine to preeclampsia spans observational cohorts, mechanistic correlation studies, and meta-analyses. The association at delivery was established in classic work by Rajkovic et al., who reported elevated homocysteine in preeclamptic nulliparas at the time of delivery [9]. Homocysteine offers biological plausibility as both a marker and mediator within this vascular paradigm. Hyperhomocysteinemia is associated with oxidative stress and impaired nitric oxide biology and can promote endothelial injury - mechanisms classically described by Loscalzo and supported by broader vascular literature [2]. Homocysteine is also linked to prothrombotic phenotypes and vascular disease risk and is modifiable by folate and vitamin B status [10]. Importantly, homocysteine normally declines during uncomplicated pregnancy [11]; therefore, an elevation in late gestation may represent failure of physiological adaptation or superimposed metabolic-endothelial stress. Powers et al. demonstrated increased plasma homocysteine in preeclampsia and correlated it with markers of endothelial activation, strengthening the argument that homocysteine is not merely an epiphenomenon [12]. A widely cited synthesis - "Mapping the theories of preeclampsia: the role of homocysteine" - positioned homocysteine as a plausible contributor to endothelial injury and placental dysfunction [13]. More recently, a meta-analysis by Zhang et al. reported an association between elevated homocysteine levels and increased preeclampsia risk [14,15].

However, predictive performance is heterogeneous. Some early-pregnancy studies report increased risk for subsequent severe disease with higher homocysteine values [16], while other cohorts did not confirm predictive utility [17]. This study was therefore carried out to address this gap by evaluating whether maternal serum homocysteine levels show a progressive increase across clearly defined severity categories of HDP, from normotensive pregnancy to non-severe preeclampsia, severe preeclampsia, and eclampsia, and to assess its potential role as a severity-linked biomarker for clinical risk stratification at presentation [5].

## Materials and methods

Study design and setting

This prospective observational cohort study was conducted in the Department of Obstetrics and Gynaecology at NRS Medical College and Hospital, Kolkata, India, over a period from November 2017 to October 2020. The study was designed to evaluate the association between maternal serum homocysteine levels and the severity spectrum of HDP in a tertiary care setting. Ethical approval was obtained from the institutional review board, and written informed consent was obtained from all participants prior to enrolment.

Study population

The sample size was calculated for comparison of mean serum homocysteine levels across four independent groups using one-way analysis of variance. Assuming a medium effect size of f = 0.25, an alpha error of 0.05, statistical power of 80%, and four study groups, the minimum required sample size was approximately 180 participants. To allow balanced group allocation and account for possible incomplete data, a total sample size of 200 participants was selected, with 50 women in each group: normotensive controls, non-severe preeclampsia, severe preeclampsia, and eclampsia. With this sample size, the study had an estimated statistical power of approximately 85% to detect a medium difference in serum homocysteine levels across the four groups.

Diagnostic criteria

Hypertensive disorders were classified using standard obstetric criteria consistent with contemporary ISSHP and ACOG frameworks.

Normotensive pregnancy was defined as systolic blood pressure < 140 mmHg and diastolic blood pressure < 90 mmHg in the absence of proteinuria or end-organ dysfunction.

Non-severe preeclampsia was defined as blood pressure ≥ 140/90 mmHg on two occasions at least four hours apart after 20 weeks of gestation, with proteinuria of ≥ 300 mg in 24 hours or equivalent dipstick positivity.

Severe preeclampsia was defined as blood pressure ≥ 160/110 mmHg or the presence of severe features, such as thrombocytopenia, renal insufficiency, impaired liver function, pulmonary edema, or neurological symptoms.

Eclampsia was defined as generalized tonic-clonic seizures occurring in a woman with preeclampsia and not attributable to other neurological causes.

Inclusion and exclusion criteria

Women were included if they had a singleton pregnancy of at least 20 weeks' gestation and fulfilled the diagnostic criteria for one of the four study groups.

Women were excluded if they had pre-existing chronic hypertension, diabetes mellitus, renal disease, hepatic disease, cardiovascular disease, multiple gestation, or known metabolic disorders affecting homocysteine metabolism, including suspected folate or vitamin B12 deficiency, although these were not biochemically measured.

Clinical assessment and data collection

Baseline demographic and clinical variables were recorded at admission, including maternal age, parity, socioeconomic status, body mass index (BMI), and blood pressure. Blood pressure was measured using a calibrated sphygmomanometer with the patient in a semi-recumbent position according to standard protocol.

Biochemical analysis

Venous blood samples were collected at admission before initiation of antihypertensive therapy, magnesium sulfate administration, or delivery-related intervention. This pre-intervention sampling was intended to reduce treatment-related confounding and reflect the biochemical state corresponding to disease severity. Serum homocysteine levels were measured by the standard laboratory assay protocol and reported in µmol/L.

Outcome measures

The primary outcome was maternal serum homocysteine concentration at admission. Secondary outcomes included mode of delivery, categorized as vaginal delivery or lower segment cesarean section, and maternal blood pressure at 12 weeks postpartum to assess resolution of the hypertensive disorder.

Statistical analysis

Data were analyzed using standard statistical software. Continuous variables were expressed as mean ± standard deviation and compared across groups using one-way analysis of variance (ANOVA). Categorical variables were expressed as frequency and percentage and compared using the chi-square test. A p-value of < 0.05 was considered statistically significant.

Effect size estimation

To improve interpretability, effect estimates such as mean differences and proportional differences were derived from summary statistics using standard parametric formulas. These calculations were performed without re-estimating p-values.

Methodological considerations

The study had several methodological strengths, including prospective data collection, equal group sizes, and biomarker sampling before therapeutic intervention. However, serum folate and vitamin B12 levels were not measured, and this may have introduced residual confounding in the interpretation of homocysteine levels.

## Results

A total of 200 pregnant women were included and equally distributed into four groups: normotensive controls (n = 50), non-severe preeclampsia (n = 50), severe preeclampsia (n = 50), and eclampsia (n = 50). Maternal age was comparable across all groups (F(3,196) = 0.80, p = 0.4936), with mean values ranging from 24.46 ± 4.58 years in the non-severe preeclampsia group to 25.96 ± 5.26 years in the eclampsia group.

Statistically significant differences in parity were observed between groups (χ²(3) = 8.46, p = 0.0375). Primigravid women accounted for 25/50 (50.0%) of the normotensive group, 29/50 (58.0%) of the non-severe preeclampsia group, 35/50 (70.0%) of the severe preeclampsia group, and 27/50 (54.0%) of the eclampsia group. Socioeconomic status distribution did not differ significantly across groups (χ²(3) = 5.66, p = 0.1297) (Table 1). Maternal BMI increased significantly with disease severity (F(3,196) = 2.71, p = 0.0461), rising from 22.95 ± 1.49 kg/m² in normotensive women to 26.88 ± 1.61 kg/m² in the eclampsia group.

**Table 1:** Baseline, clinical, and biochemical characteristics of the study groups. ANOVA results are reported as F (df₁, df₂). Chi-square results are reported as χ² (df).

Variable	Normotensive (n=50)	Non-severe preeclampsia (n=50)	Severe preeclampsia (n=50)	Eclampsia (n=50)	p-value	Statistic
Age (years), mean ± SD	25.00 ± 4.26	24.46 ± 4.58	25.28 ± 5.51	25.96 ± 5.26	0.4936	F(3,196) = 0.80
Primigravida, n (%)	25 (50.0)	29 (58.0)	35 (70.0)	27 (54.0)	0.0375	χ²(3) = 8.46
Lower socioeconomic status, n (%)	38 (76.0)	45 (90.0)	40 (80.0)	45 (90.0)	0.1297	χ²(3) = 5.66
BMI (kg/m²), mean ± SD	22.95 ± 1.49	24.59 ± 1.63	26.44 ± 2.04	26.88 ± 1.61	0.0461	F(3,196) = 2.71
SBP on admission (mmHg), mean ± SD	126.16 ± 6.06	146.32 ± 4.63	167.40 ± 5.77	176.48 ± 6.61	<0.001	F(3,196) = 412.6
DBP on admission (mmHg), mean ± SD	77.32 ± 5.31	97.60 ± 5.00	114.84 ± 3.98	114.56 ± 3.13	<0.001	F(3,196) = 521.4
Serum homocysteine (µmol/L), mean ± SD	12.04 ± 2.34	18.10 ± 1.01	21.25 ± 2.03	26.74 ± 6.58	0.0251	F(3,196) = 3.21
SBP at 12 weeks postpartum (mmHg), mean ± SD	125.88 ± 5.99	126.56 ± 6.06	127.04 ± 4.36	127.40 ± 4.52	0.5112	F(3,196) = 0.77
DBP at 12 weeks postpartum (mmHg), mean ± SD	76.20 ± 4.95	78.40 ± 3.83	77.88 ± 5.20	77.32 ± 4.58	0.1094	F(3,196) = 2.02

Systolic and diastolic blood pressures on admission were significantly higher in hypertensive groups compared to normotensive controls. Mean systolic blood pressure increased progressively across groups (F(3,196) = 412.6, p < 0.001), from 126.16 ± 6.06 mmHg in normotensive women to 176.48 ± 6.61 mmHg in eclampsia. Mean diastolic blood pressure also increased significantly (F(3,196) = 521.4, p < 0.001), from 77.32 ± 5.31 mmHg to 114.56 ± 3.13 mmHg (Table 1).

Maternal serum homocysteine levels increased significantly with advancing disease severity (F(3,196) = 3.21, p = 0.0251). Mean values were 12.04 ± 2.34 µmol/L in normotensive women, 18.10 ± 1.01 µmol/L in non-severe preeclampsia, 21.25 ± 2.03 µmol/L in severe preeclampsia, and 26.74 ± 6.58 µmol/L in eclampsia (Table 1).

The mode of delivery differed significantly across groups (χ²(9) = 68.4, p < 0.001). Lower segment cesarean section rates increased with disease severity, from 7/50 (14.0%) in normotensive women to 14/50 (28.0%) in non-severe preeclampsia, 35/50 (70.0%) in severe preeclampsia, and 40/50 (80.0%) in eclampsia. Vaginal delivery rates declined from 30/50 (60.0%) in normotensive women to 3/50 (6.0%) in eclampsia (Table 2).

**Table 2:** Mode of delivery across the severity spectrum of hypertensive disorders of pregnancy. Overall comparison of the mode of delivery across disease severity groups showed a statistically significant association (chi-square value of 68.4 with 9 degrees of freedom; p < 0.001). p < 0.001 for difference across groups. LSCS: Lower segment caesarean section

Mode of delivery	Normotensive (n=50)	Non-severe preeclampsia (n=50)	Severe preeclampsia (n=50)	Eclampsia (n=50)	Total (n=200)
Forceps, n (%)	7 (14.0)	5 (10.0)	5 (10.0)	4 (8.0)	21 (10.5)
LSCS, n (%)	7 (14.0)	14 (28.0)	35 (70.0)	40 (80.0)	96 (48.0)
Vaginal delivery, n (%)	30 (60.0)	29 (58.0)	6 (12.0)	3 (6.0)	68 (34.0)
Ventouse, n (%)	6 (12.0)	2 (4.0)	4 (8.0)	3 (6.0)	15 (7.5)

At 12 weeks postpartum, systolic and diastolic blood pressures were comparable across all groups, with no statistically significant differences (SBP: F(3,196) = 0.77, p = 0.5112; DBP: F(3,196) = 2.02, p = 0.1094).

## Discussion

**Figure 1:**
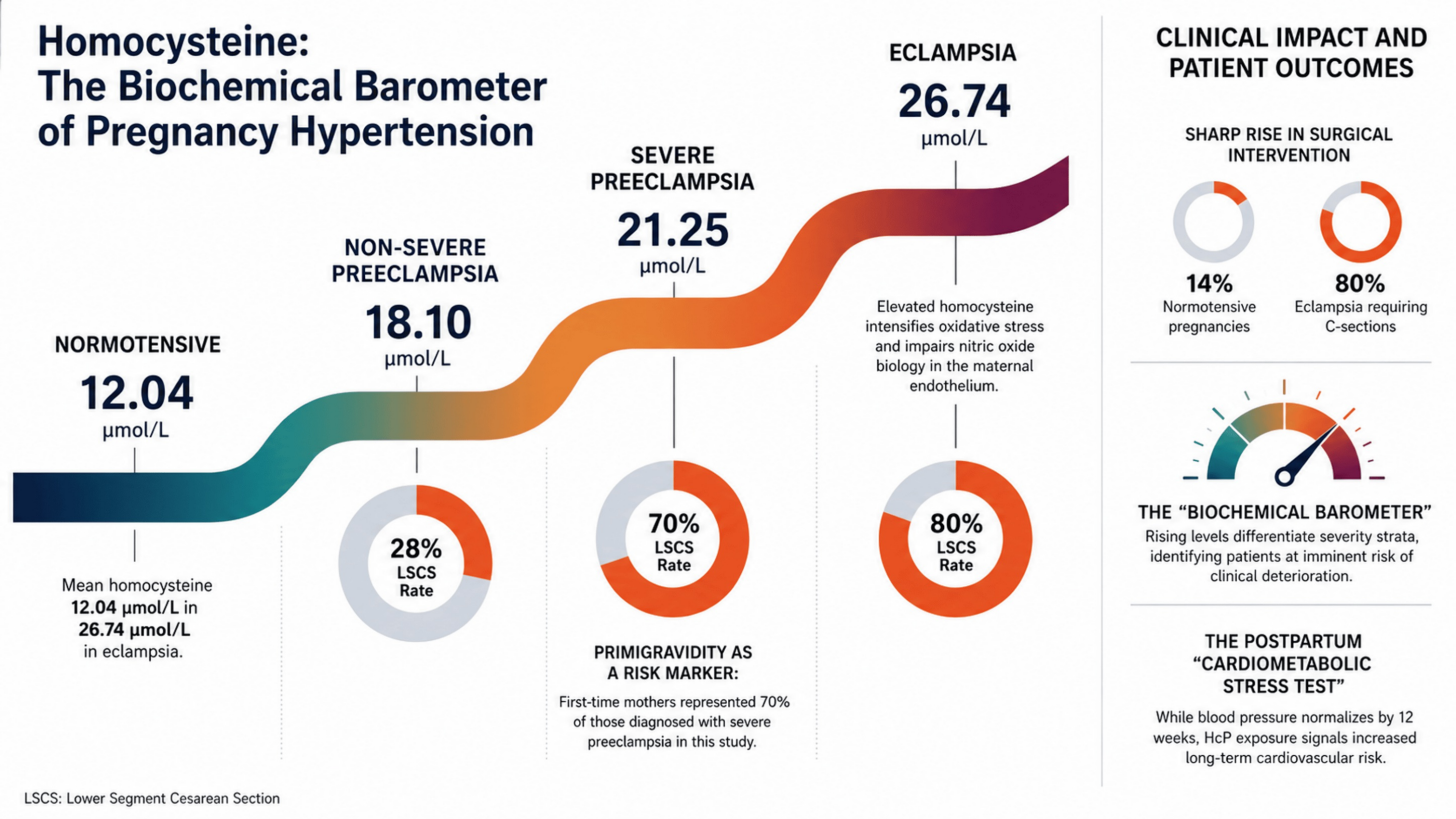
The progressive relationship between maternal serum homocysteine levels and the severity of hypertensive disorders of pregnancy (HDP), conceptualized as a "biochemical barometer" of disease progression. The image was designed by the authors using Canva (Canva Pty Ltd., Sydney, Australia) as a visual illustration of the observed stepwise increase in maternal serum homocysteine levels across the severity spectrum of hypertensive disorders of pregnancy.

In this prospective observational study of 200 pregnant women stratified across the full clinical spectrum of HDP, we observed a significant and progressive increase in maternal serum homocysteine concentrations with advancing disease severity. Mean homocysteine levels rose from normotensive controls to non-severe preeclampsia and severe preeclampsia and were the highest in eclampsia, demonstrating a clear dose-response relationship (p = 0.0251) (Figure 1).

Baseline maternal characteristics were largely comparable between groups, with no significant difference in age. However, primigravidity was significantly more prevalent in severe disease categories (p = 0.0375), supporting its established role as a risk factor for preeclampsia. In addition, maternal BMI increased with disease severity (p = 0.0461), suggesting a contributory role of metabolic and inflammatory factors.

As expected, systolic and diastolic blood pressures increased significantly across the disease spectrum (p < 0.001), confirming appropriate clinical stratification. Importantly, the mode of delivery differed markedly between groups, with a substantially higher rate of caesarean section in severe preeclampsia (70%) and eclampsia (80%) compared with normotensive and non-severe groups (p < 0.001), reflecting increased obstetric intervention in advanced disease.

At 12 weeks postpartum, blood pressure values were comparable across all groups, indicating resolution of the acute hypertensive state, although the antecedent severity raises concerns regarding long-term cardiovascular risk. Taken together, these findings demonstrate that maternal serum homocysteine is strongly associated with both the presence and severity of HDP and may serve as a clinically relevant biomarker for disease stratification at presentation.

The study design has several strengths: equal distribution across clinically meaningful severity strata (including eclampsia) and inclusion of 12-week postpartum blood pressure follow-up, enabling separation of acute, pregnancy-limited haemodynamic effects from longer-term persistence.

Key limitations include modest sample size (n=200) for advanced modelling, absence of folate/B12/renal function-adjusted analyses (major determinants of homocysteine), and reliance on single time-point homocysteine measurement on admission (unknown within-person trajectory). Additionally, without individual-level homocysteine distributions, receiver operating characteristic (ROC)-based thresholds for triage escalation cannot be derived.

Generalisability is likely the strongest to tertiary-care populations similar to those seen in our hospital and to comparable academic settings, such as the West Bengal University of Health Sciences [13] framework.

This dataset demonstrates a clinically intuitive and statistically supported pattern: maternal serum homocysteine rises progressively from normotensive pregnancy through non-severe and severe preeclampsia and peaks in eclampsia. This aligns with the "endothelial convergence" concept in which multiple upstream insults (placental malperfusion, antiangiogenic signalling, and inflammatory activation) culminate in maternal vascular dysfunction [7].

Mechanistically, homocysteine may intensify vascular injury via oxidative stress and impaired nitric oxide signalling phenotypes with direct plausibility in preeclampsia's vasoconstrictive and prothrombotic milieu. The observed gradient also resonates with earlier clinical evidence: Rajkovic et al.'s delivery-time observation of elevated homocysteine in preeclampsia [9] and Powers et al.'s correlation between homocysteine and endothelial activation markers [12]. Contemporary synthesis supports risk association at the population level (meta-analysis evidence) [15].

The "Hormuz Valve" of pregnancy (a clinically useful analogy for escalation risk) can be used cautiously as an explanatory model rather than a validated doctrine: as disease severity advances, vascular reserve narrows like a chokepoint, and homocysteine behaves as a pressure gauge for endothelial/placental stress. In this cohort, mean homocysteine differentiates severity strata with clinically meaningful separations (e.g., severe vs non-severe mean difference ≈ 3.15 µmol/L; eclampsia vs severe ≈ 5.49 µmol/L). This supports the hypothesis that rising homocysteine could serve as an adjunct severity biomarker for imminent deterioration - particularly relevant in overloaded obstetric units where rapid triage decisions are required.

However, literature cautions that predictive utility varies by gestational timing and phenotype; some cohorts did not find early homocysteine to be a robust predictor [17]. Therefore, the most defensible near-term clinical use is not "prediction" of preeclampsia de novo but risk-stratified escalation among clinically established HDP, as examined here.

BMI increased significantly with severity, with an absolute mean difference of ~3.9 kg/m² between eclampsia and controls. This is consistent with broader epidemiology: pre-pregnancy obesity is strongly associated with preeclampsia risk and is increasingly associated with severe-feature phenotypes, likely via insulin resistance, chronic inflammation, oxidative stress, and endothelial activation [18].

The primigravida excess in severe preeclampsia (70%) is also consistent with established risk literature. A systematic review by Kate Duckitt [19] reported substantially increased preeclampsia risk in nulliparous women, reinforcing parity as a major baseline risk marker [20]. This parity association is also embedded in clinical risk frameworks used to guide prophylaxis (e.g., aspirin eligibility) [21]. In the present dataset, primigravidity was not uniformly highest in eclampsia, which may reflect referral patterns, timing of presentation, or competing pathways (e.g., multiparous women with higher baseline cardiometabolic burden).

Lower segment caesarean section (LSCS) frequency increased dramatically with severity (80% in eclampsia). This aligns with practice realities: deteriorating maternal condition, fetal compromise, unfavourable cervix, and need for expedited delivery frequently drive operative delivery in severe HDP and eclampsia. Guidance emphasises delivery as definitive management for established severe disease, although the route should remain individualised.

Blood pressure normalised by 12 weeks postpartum in all groups (SBP and DBP both non-significant), consistent with the concept that many HDP phenotypes resolve by 6-12 weeks [22]. However, the postpartum finding should be positioned carefully: HDP is increasingly recognised as a cardiometabolic stress test that unmasks future cardiovascular vulnerability. Meta-analytic evidence indicates that preeclampsia is associated with substantially increased long-term risk of hypertension, ischaemic heart disease, stroke, and heart failure [23]. This is reinforced in cardiovascular guidance and contemporary postpartum hypertension reviews from the American Heart Association ecosystem [24].

Because homocysteine is influenced by folate and B12 status, lack of micronutrient measurement limits causal interpretation [25]. Importantly, trials attempting to prevent preeclampsia via homocysteine modulation have not uniformly succeeded; for example, high-dose folic acid beyond the first trimester did not prevent preeclampsia in high-risk women in the FACT trial reported by Wen [19]. This suggests that homocysteine may be a downstream marker of vascular stress in some phenotypes or that intervention timing and biological pathways are more complex than simple lowering of the biomarker.

## Conclusions

Maternal serum homocysteine demonstrates a clear and progressive rise across the spectrum of hypertensive disorders of pregnancy, from normotensive states to eclampsia. This consistent stepwise escalation not only reinforces its biological link to endothelial dysfunction but also positions homocysteine as a clinically meaningful indicator of disease severity at presentation.

From a clinical perspective, homocysteine has the potential to function as a practical and accessible biomarker for early risk stratification, particularly in high-burden obstetric settings where timely recognition of severe disease is critical. Its incorporation into routine assessment could strengthen triage decisions, enable more targeted surveillance, and support earlier intervention. While further large-scale validation is required, these findings highlight a promising step toward more precise, biomarker-driven obstetric care with the potential to improve maternal outcomes.
